# Supplementary material for: Structure determination from single molecule X-ray scattering with three photons per image
Source: Nat Commun. 2018 Jun 18;9:2375. doi: 10.1038/s41467-018-04830-4 (PMC6006178; doi:10.1038/s41467-018-04830-4)
Supplement: Supplementary file 1 — Supplementary Information [file 41467_2018_4830_MOESM1_ESM.pdf]

Supplementary Material: Structure determination from single molecule X-ray scattering with three photons per image

Benjamin von Ardenne,[*] Martin Mechelke,[†] and Helmut Grubmüller[‡]

Department of Theoretical and Computational Biophysics,

Max Planck Institute for Biophysical Chemistry Göttingen,

Am Fassberg 11, 37077 Göttingen, Germany

[*] bardenn@mpibpc.mpg.de

[†] present address: martin.mechelke@gmail.com

[‡] Corresponding Author: hgrubmu@mpibpc.mpg.de

SUPPLEMENTARY NOTE 1: DERIVATION OF THE THREE-PHOTON CORRELATION EXPRESSED IN SPHERICAL HARMONICS

Here we derive the three-photon correlation $t(k_1, k_2, k_3, \alpha, \beta)$, as defined in Fig. 1b of the main text, as a function of the three-dimensional intensity in Fourier space, $I(\mathbf{k}) = |\mathcal{FT}[\rho(\mathbf{x})]|^2$, which is expanded using a spherical harmonics basis. The following derivation follows Kam [1], but further generalizes it to the full three-photon correlation.

The triple correlation $t(k_1, k_2, k_3, \alpha, \beta)$ is the orientational average $\langle\rangle_\omega$ of the product between three intensities $I(\mathbf{k})$ that lie on the intersection between the Ewald sphere and the 3D Fourier density,

$$t(k_1, k_2, k_3, \alpha, \beta)_{I(\mathbf{k})} = \langle I_\omega \left(\mathbf{k}_1^\star(k_1, 0)\right) \cdot I_\omega \left(\mathbf{k}_2^\star(k_2, \alpha)\right) \cdot I_\omega^* \left(\mathbf{k}_3^\star(k_3, \beta)\right)\rangle_\omega . \quad (1)$$

Here, without loss of generality, the three vectors $\mathbf{k_1}^\star$, $\mathbf{k_2}^\star$ and $\mathbf{k_3}^\star$ are the projection onto the Ewald sphere of the three photons $\mathbf{k_1} = (k_1, 0, 0)$, $\mathbf{k_2} = k_2(\cos\alpha, \sin\alpha, 0)$ and $\mathbf{k_3} = k_3(\cos\beta, \sin\beta, 0)$ in the detector plane. The photons positions are chosen as one arbitrary realization of the triplet $(k_1, k_2, k_3, \alpha, \beta)$, characterized by the angles α and β between the vectors and distances to the detector k_1, k_2 and k_3, respectively (see Fig. 1b in main text). For the orientational average $\langle\rangle_\omega$ it is assumed that in the experiment the orientation of the molecule is unknown and uniformly sampled. Note that the orientational average can either be expressed as an average over all rotations of $I_\omega(\mathbf{k})$ for fixed $\mathbf{k}_{1,2,3}$ (our approach) or as an average over all rotations of the vectors $\mathbf{k}_{1,2,3,\omega}$ for a fixed $I(\mathbf{k})$.

Next, $I(\mathbf{k})$ is decomposed into spherical shells with radius k and each shell is expanded

using a spherical harmonics basis [2],

$$I\left(\mathbf{k}\right)=\sum_{lm}A_{lm}\left(k\right)Y_{lm}\left(\theta,\varphi\right). \tag{2}$$

The coefficients $A_{lm}(k)$ describe the intensity function on the respective shells and are non-zero only for even $l \in \{0,2,4,...,L\}$ because of the symmetry of $I(\mathbf{k}) = I(-\mathbf{k})$ (Friedel's law). In this description, a 3D Euler rotation ω of $I(\mathbf{k})$ is expressed by transforming the spherical harmonics coefficients according to $A_{lm}^{\mathrm{rot}}(k) = \sum_{mm'} D_{mm'}^{l} A_{lm'}^{\mathrm{unrot}}(k)$, using the rotation operators $D_{m'm}^{l}$ which are composed of elements of the Wigner D-matrix as defined, e.g., in Ref. [2], yielding the rotated intensity,

$$I_{\omega}\left(\mathbf{k}\right)=\sum_{lmm'}A_{lm}\left(k\right)Y_{lm'}\left(\theta,\varphi\right)D_{m'm}^{l}\left(\omega\right). \tag{3}$$

Inserting the spherical harmonics expansion of the rotated intensity $I_{\omega}\left(\mathbf{k}\right)$, evaluated at positions $\mathbf{k}_1^{\star}$, $\mathbf{k}_2^{\star}$ and $\mathbf{k}_3^{\star}$ on the Ewald sphere ($\theta_i = \cos^{-1}(\frac{k_i\lambda}{4\pi})$), into the expression for the three-photon correlation, Supplementary Eq. 1, yields

$$\begin{aligned}t(k_1,k_2,k_3,\alpha,\beta)_{\{A_{lm}(k)\}} = &\sum_{l_1\,l_2\,l_3}\sum_{m_1\,m_2\,m_3}\sum_{m_1'\,m_2'\,m_3'} A_{l_1m_1}\left(k_1\right)A_{l_2m_2}\left(k_2\right)A_{l_3m_3}^{*}\left(k_3\right)\\ &\times Y_{l_1m_1'}\left(\theta_1(k_1),0\right)\cdot Y_{l_2m_2'}\left(\theta_2(k_2),\alpha\right)\cdot Y_{l_3m_3'}^{*}\left(\theta_3(k_3),\beta\right)\\ &\times\left\langle D_{m_1m_1'}^{l1}\cdot D_{m_2m_2'}^{l2}\cdot D_{m_3m_3'}^{l3}\right\rangle_{\omega},\end{aligned} \tag{4}$$

such that the orientational average only involves the elements of the Wigner D-matrix $D_{mm'}^{l}$. Using the Wigner-3j symbols $\begin{pmatrix} l_1 & l_2 & L \\ m_1 & m_2 & -M \end{pmatrix}$ [3], the product of two rotation elements $D_{mm'}^{l}$ reads

$$D^{l_1}_{m_1 m'_1} D^{l_2}_{m_2 m'_2} = \sum_{L=|l_1-l_2|}^{l_1+l_2} \sum_{MM'} (2L+1)\,(-1)^{M-M'} \times \begin{pmatrix} l_1 & l_2 & L \\ m_1 & m_2 & -M \end{pmatrix} \begin{pmatrix} l_1 & l_2 & L \\ m'_1 & m'_2 & -M' \end{pmatrix} D^L_{MM'}. \tag{5}$$

With the orthogonality theorem for orientational averages of the product of two Wigner D operators,

$$\left\langle D^L_{MM'} D^{l3*}_{m_3 m'_3} \right\rangle_\omega = \frac{1}{2L+1} \delta_{l_3 L} \delta_{m_3 M} \delta_{m'_3 M'}, \tag{6}$$

the three-photon correlation finally reads

$$t(k_1, k_2, k_3, \alpha, \beta)_{\{A_{lm}(k)\}} = \sum_{l_1\, l_2\, l_3} \sum_{m_1\, m_2\, m_3} A_{l_1 m_1}(k_1)\, A_{l_2 m_2}(k_2)\, A^*_{l_3 m_3}(k_3) \times \begin{pmatrix} l_1 & l_2 & l_3 \\ m_1 & m_2 & -m_3 \end{pmatrix} \sum_{m'_1\, m'_2\, m'_3} (-1)^{m_3 - m'_3} \begin{pmatrix} l_1 & l_2 & l_3 \\ m'_1 & m'_2 & -m'_3 \end{pmatrix} \times Y_{l_1 m'_1}(\theta_1(k_1), 0)\, Y_{l_2 m'_2}(\theta_2(k_2), \alpha)\, Y^*_{l_3 m'_3}(\theta_3(k_3), \beta)\,. \tag{7}$$

This expression only involves sums of products of three spherical harmonics coefficients $A_{lm}(k)$ with known Wigner-3j symbols and spherical harmonics basis functions $Y_{lm}(\theta, \varphi)$.

SUPPLEMENTARY NOTE 2: EFFICIENT COMPUTATION OF THE THREE-PHOTON CORRELATION

Our method requires the fast evaluation of the three-photon correlation for a proposed set of spherical harmonics coefficients $\{A_{lm}(k)\}$. To that end, we vectorized Supplementary

Eq. 7 as follows

$$t(k_1,k_2,k_3,\alpha,\beta)_{\{A_{lm}(k)\}} = \sum_{l_1\,l_2\,l_3} A^S(l_1,l_2,l_3,k_1,k_2,k_3) \times [\mathbf{p}(l_1,l_2,l_3,k_1,k_2,k_3)\cdot\mathbf{b}(l_1,l_2,l_3,\alpha,\beta)] = \mathbf{p}_A(k_1,k_2,k_3)\cdot\mathbf{b}(\alpha,\beta). \quad (8)$$

using

$$A^S(l_1,l_2,l_3,k_1,k_2,k_3) = \sum_{m_1\,m_2\,m_3} A_{l_1m_1}(k_1)\,A_{l_2m_2}(k_2)\,A^*_{l_3m_3}(k_3) \times(-1)^{m_3}\begin{pmatrix} l_1 & l_2 & l_3 \\ m_1 & m_2 & -m_3 \end{pmatrix}. \quad (9)$$

The vector $\mathbf{p}$ contains the k-dependant Legendre polynomials and $\mathbf{b}$ contains the α,β-dependant complex exponential of the spherical harmonics together with the Wigner-3j symbol which are non-zero only for $m_1+m_2+m_3=0$ and $|l_1-l_2|\leq l_3\leq l_1+l_2$. The products between A^S and the entries of $\mathbf{p}$ are denoted $\mathbf{p}_A$. The entire three-photon correlation $\mathbf{T}$ is then calculated by the matrix product

$$\mathbf{T} = \mathbf{P}_A\cdot\mathbf{B}, \quad (10)$$

with the matrix $\mathbf{P}_A \in \mathbb{R}^{B\times K^3}$, the matrix $\mathbf{B}\in\mathbb{R}^{N^2\times B}$ and the full three-photon correlation matrix $\mathbf{T}\in\mathbb{R}^{N^2\times K^3}$ with the entries $T_{ij} = t((k_1,k_2,k_3)(i),(\alpha,\beta)(j))$. Here, we denote the number of non-zero index combinations $(l_1,l_2,l_3,m_1,m_2,m_3)$ as B and the number of discrete angles $\alpha,\beta\in[0,\pi]$ in one dimension as N, as further described in Supplementary Note 5.

This vectorized expression can be calculated with a high degree of parallelism, but nevertheless becomes the limiting factor in the computation. In particular, the number B of three-photon basis functions $f(l_1,l_2,l_3,m_1,m_2,m_3,\alpha,\beta)$ grows quickly with $B\sim L^4$ (e.g.,

$B = 11,841$ for $L = 10$ and $B = 163,153$ for $L = 18$) and the time to calculate the full three-photon correlation matrix $\mathbf{T}$ therefore scales with $K \cdot (K+1) \cdot (K+2)/6 \cdot L^4$. The number of shells K and angular resolution L required to resolve an intensity for a given resolution scales linear with the object diameter and the complexity therefore scales approx. n^7 with the ratio n between diameter and resolution or $M^{2.33}$ with M the molecular weight. At the same time, the computational cost is independent of the number of images or number of photons per image, as these numbers only determine the time to assemble the three-photon histogram. See Supplementary Note 7 on how the scaling of the computational complexity affects our choice of spherical harmonics parameters.

In our implementation, we calculated both the entries of $\mathbf{P}_A$ and the matrix multiplication for $\mathbf{T}$ with a custom CUDA kernel, which significantly improved ($>$ 100x) the performance over CPU-based implementations and thus rendered the optimization computationally tractable.

SUPPLEMENTARY NOTE 3: IMPLEMENTATION OF THE SPHERICAL HARMONICS EXPANSION

All Fast Spherical Harmonics Transformations were performed using the S2Kit framework (http://www.cs.dartmouth.edu/∼geelong/sphere) [4, 5]. The same spherical harmonics expansion order L was used for all shells. For the structure determination, $L = 18$ was used, which yields $(2L)^2 = 1296$ sample points on the sphere with an even sampling in $\phi \in [0, 2\pi]$ and $\theta \in [-\pi/2, \pi/2]$ direction. The angular resolution of the expansion is $\Delta\theta = \pi/(2L)$ or $\Delta\varphi = 2\pi/(2L)$ respectively which in our case for $L = 18$ corresponds to an angular resolution of $\Delta\theta = 5.0°$ in longitude direction and $\Delta\varphi = 10.0°$ in latitude direction. The density $\rho(\mathbf{x})$, expanded with a spherical harmonics basis, was Fourier transformed by applying the

spherical Bessel transform (Hankel transform) to the coefficients according to Ref. [6–8] All Wigner matrices were calculated as described in Ref. [9, 10] and the absolute square of the Fourier density was calculated according to Ref. [11] by transforming the coefficients directly.

SUPPLEMENTARY NOTE 4: IMPLEMENTATION DETAILS OF THE MONTE CARLO OPTIMIZATION

The random rotations $\left\{\mathbf{U}_l \in R^{2l+1\times 2l+1}\right\}$ were generated using QR-decompositions of normal-distributed matrices as described by Mezzadri [12]. The rotational variations $\mathbf{\Delta}_l\left(\beta\right)$ were calculated via the basis transformation

$$\mathbf{\Delta}_l\left(\beta\right) = \mathbf{R}_l \mathbf{S}_l\left(\beta\right) \mathbf{R}_l^{-1} \tag{11}$$

with

$$\mathbf{S}_l\left(\beta\right) = \begin{pmatrix} \cos\left(\beta\right) & -\sin\left(\beta\right) & 0 & ... & 0 \\ \sin\left(\beta\right) & \cos\left(\beta\right) & 0 & ... & 0 \\ 0 & 0 & I_{2l+1-2} & & \\ ... & ... & & & \\ 0 & 0 & & & \end{pmatrix} \tag{12}$$

and random rotation matrices $\mathbf{R}_l$ [13]. Here, sub-matrix $\mathbf{I}_{2l-1}$ in $\mathbf{S}_l$ is a $2l-1$-dimensional unity matrix.

By using the small rotational variations $\mathbf{\Delta}_l\left(\beta\right)$, the SO(n) is sampled ergodically. Approximately $[1/(2-2\cos(\beta))]n\cdot\log(n)$ steps are necessary to achieve sufficient sampling according to Ref. [13]. For the largest search space of $L = 18$ with a rotation dimension of $n = 37$ ($n = 2L+1$) and a minimum stepsize of $\beta = 0.025$ rad, 213,777 steps were required to sample rotations in $SO(37)$ sufficiently dense. To ensure that the search space is exhaustively explored, we aimed at an optimization length of over 200,000 Monte Carlo

steps. To this end, a time constant for the temperature decrease of $\tau = 50,000$ steps was chosen. The initial temperature T_{init} was calculated as 10% of the standard deviation of the energy within 50 random steps away from the starting structure using the initial stepsizes. Further, we used a factor $\mu = 1.01$ for the adaptive stepsizes. The hierarchical approach was implemented by distributing the initial stepsizes according to $\beta(l) = (l-1)\pi$ such that spherical harmonics coefficients with larger expansion orders l are always varied with a larger stepsize $\beta(l)$ than coefficients with lower orders. Supplementary Figure 1 outlines the steps of the structure determination using a Monte Carlo simulated annealing method.

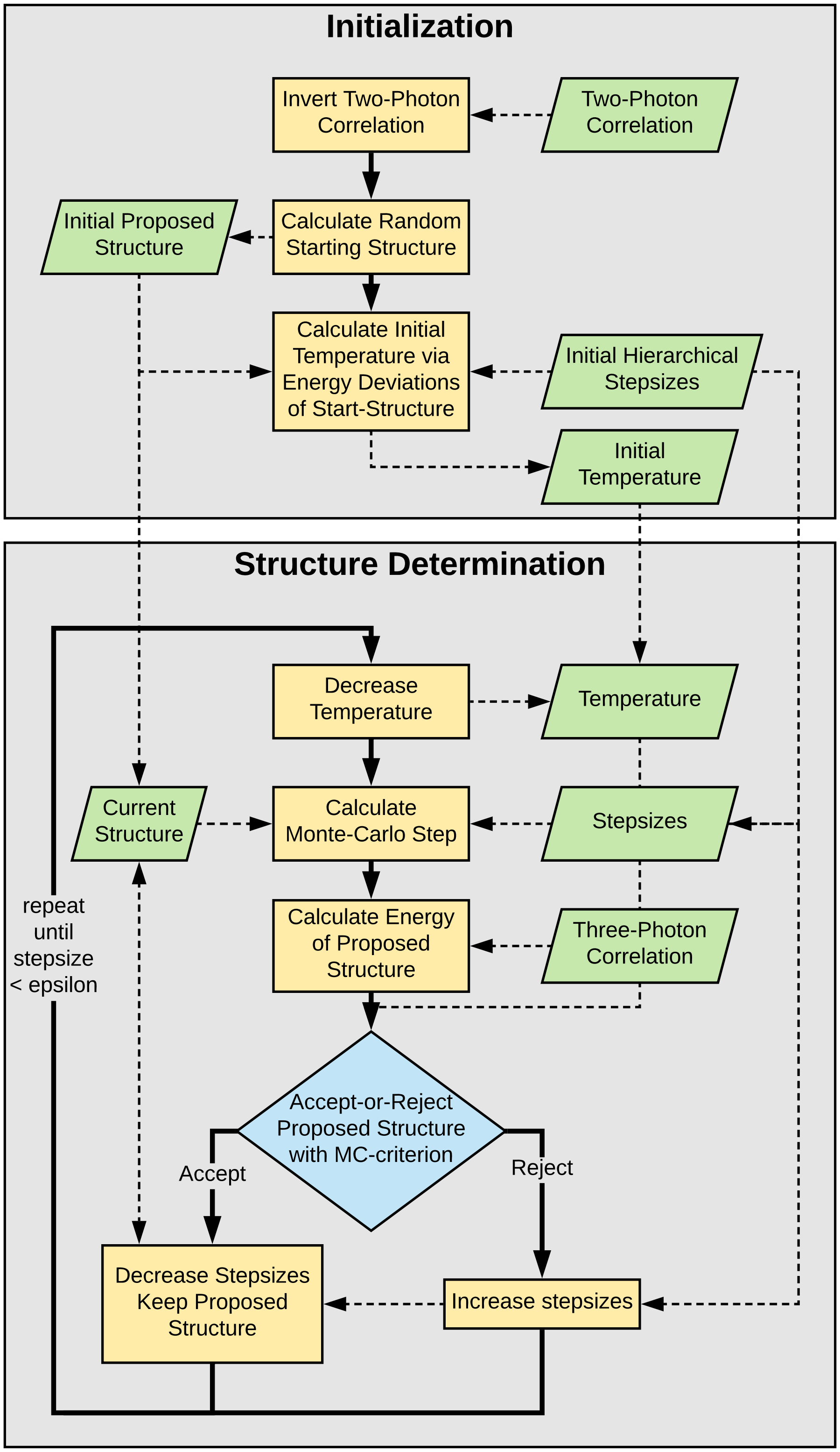
Initialization
Invert Two-Photon Correlation
Two-Photon Correlation
Initial Proposed Structure
Calculate Random Starting Structure
Calculate Initial Temperature via Energy Deviations of Start-Structure
Initial Hierarchical Stepsizes
Initial Temperature
Structure Determination
Decrease Temperature
Temperature
Current Structure
Calculate Monte-Carlo Step
Stepsizes
Calculate Energy of Proposed Structure
Three-Photon Correlation
repeat until stepsize < epsilon
Accept-or-Reject Proposed Structure with MC-criterion
Accept
Reject
Decrease Stepsizes Keep Proposed Structure
Increase stepsizes

Supplementary Figure 1: Structure Determination Flowchart

Flowchart outlining the simulated annealing Monte Carlo structure optimization algorithm. The random starting structure is calculated from the inversion of the two-photon correlation. The initial temperature of the simmulated annealing scheme is calculated from the standard deviation of the energy within 50 random steps away from the starting structure given the initial stepsize. During the Monte Carlo run, the temperature is exponentially decreased and the stepsizes for individual l are modified in each step (decrease by fixed stepsize-factor if step accepted, increase by fixed stepsize-factor if step rejected). The Monte Carlo run is finished if the system has fully cooled down and the stepsizes fall below $\epsilon_{\text{stepsize}}$.

SUPPLEMENTARY NOTE 5: EFFICIENT COMPUTATION OF THE ENERGY USING HISTOGRAMS

Calculating the probability from Eq. 4 (and energy in the Monte Carlo scheme) is computationally expensive due to the typically large number of triples T. We therefore approximated this product by grouping triplets with similar angles α, β and distances k into bins and calculated the function $t(k_1, k_2, k_3, \alpha, \beta)$ for each bin only once, denoted $t_{k_1,k_2,k_3,\alpha,\beta}$, thus markedly reducing the number of function evaluations to the number of bins. To improve the statistics for each bin, the intrinsic symmetry of the triple correlation function was also used. In particular, all triplets were mapped into the sub-region of the triple correlation that satisfies $k_1 \geq k_2 \geq k_3$. Special care was taken to correct for the fact that triplets with $k_1 = k_2 \neq k_3$ or $k_1 \neq k_2 = k_3$ or $k_1 = k_3 \neq k_2$ occur 3 times more often than $k_1 = k_2 = k_3$ and triplets

with $k_1 \neq k_2 \neq k_3$ occur 6 times more often. To compensate for different binsizes, each bin was normalized by the factor $k_1 k_2 k_3$.

In our study, the two-photon and three-photon correlations were histogrammed using sets of scattering images ranging from 1.3×10^6 to 3.3×10^9 images with an average of 10 photons per shot. We further used $K_{\max} = 38$ shells and $N = 32$ $(\Delta\alpha, \Delta\beta = 5.6^\circ)$ as bin sizes in correlation space. At the end of this Note the choice for number of shells $K_{\max}$ and its impact on the resolution is discussed. In this work, the α and β discretization was varied e.g., to $N = 48$ but without an increase in the resolution of the retrieved structures, indicating that $N = 32$ is sufficiently large.

The above histogramming, required us to calculate the probability p differently. In the triplet histogram $\{n_{k_1,k_2,k_3,\alpha,\beta}\}$, the intensity is integrated over different shell volumes with width Δk each. Depending on the fluctuation of the intensity within these volumes, this leads to different integration errors for different (k_1, k_2, k_3)-combinations. However, this error decreases with smaller shell distances Δk.

To avoid this error, we compared the intensities only by the expected (α, β)-distribution of the triplets, omitting the expected relative number of triplets per (k_1, k_2, k_3)-combination. Hence, the probability p from Eq. 4 was calculated as

$$p\left(\{n(k_1, k_2, k_3, \alpha, \beta)\} \,|\, \{A_{lm}(k)\}\right) = \prod_{k_1,k_2,k_3} \prod_{\alpha,\beta} \left(\tilde{t}_{k_1,k_2,k_3,\alpha,\beta}\right)^{\tilde{n}_{k_1,k_2,k_3,\alpha,\beta}}, \tag{13}$$

normalizing the probabilities

$$\tilde{t}_{k_1,k_2,k_3,\alpha,\beta} = \frac{t_{k_1,k_2,k_3,\alpha,\beta}}{\sum_{\alpha,\beta} t_{k_1,k_2,k_3,\alpha,\beta}} \tag{14}$$

and histogram counts

$$\tilde{n}_{k_1,k_2,k_3,\alpha,\beta} = \frac{n_{k_1,k_2,k_3,\alpha,\beta}}{\sum_{\alpha,\beta} n_{k_1,k_2,k_3,\alpha,\beta}}, \tag{15}$$

for each (k_1, k_2, k_3)-combination individually. Note that the radial shape of the intensity is already encoded in the two-photon correlation.

SUPPLEMENTARY NOTE 6: DETAILS ON THE INVERSION OF THE TWO-PHOTON CORRELATION

In the inversion of the two-photon correlation, the maximum L which can be extracted, corresponding to the angular resolution of the intensity model, scales with the number of shells $K_{\max}$ (or the inverse of the shell spacing Δk respectively) used for the two-photon inversion ($l \leq L \leq K_{\max}/2$). A rotation in dimension D has $D(D-1)/2$ free angles and for $D = 2l + 1$ the sum over $2l^2 + l$ free angles for $l \in \{2, 4, ..., L\}$ yields $\frac{1}{3}(L^3 + \frac{15}{4}L^2 + \frac{7}{2}L)$ total unknown angles.

The numerical implementation of the inversion was calculated from the doublet histogram, which itself was collected in analogy to the triplet histogram (as described later in this Note). Also note here that doublets with $k_1 \neq k_2$ occur twice as often. The coefficients A^0_{lm} are retrieved as real values, all calculations are in real spherical harmonics coefficients corresponding to a real spherical harmonics basis [2, 9, 10].

SUPPLEMENTARY NOTE 7: CHOICE OF OPTIMAL SPHERICAL HARMONICS PARAMETERS

Three parameters of the spherical harmonics expansion and the histogramming control the resolution of the determined structure. First, for a maximum wave number $k_{\max}$ up to which sufficient signal is detected, the number of shells $K_{\max}$ that is used in the inversion of the two-photon correlation can be chosen freely. The choice of $K_{\max}$ determines both the shell

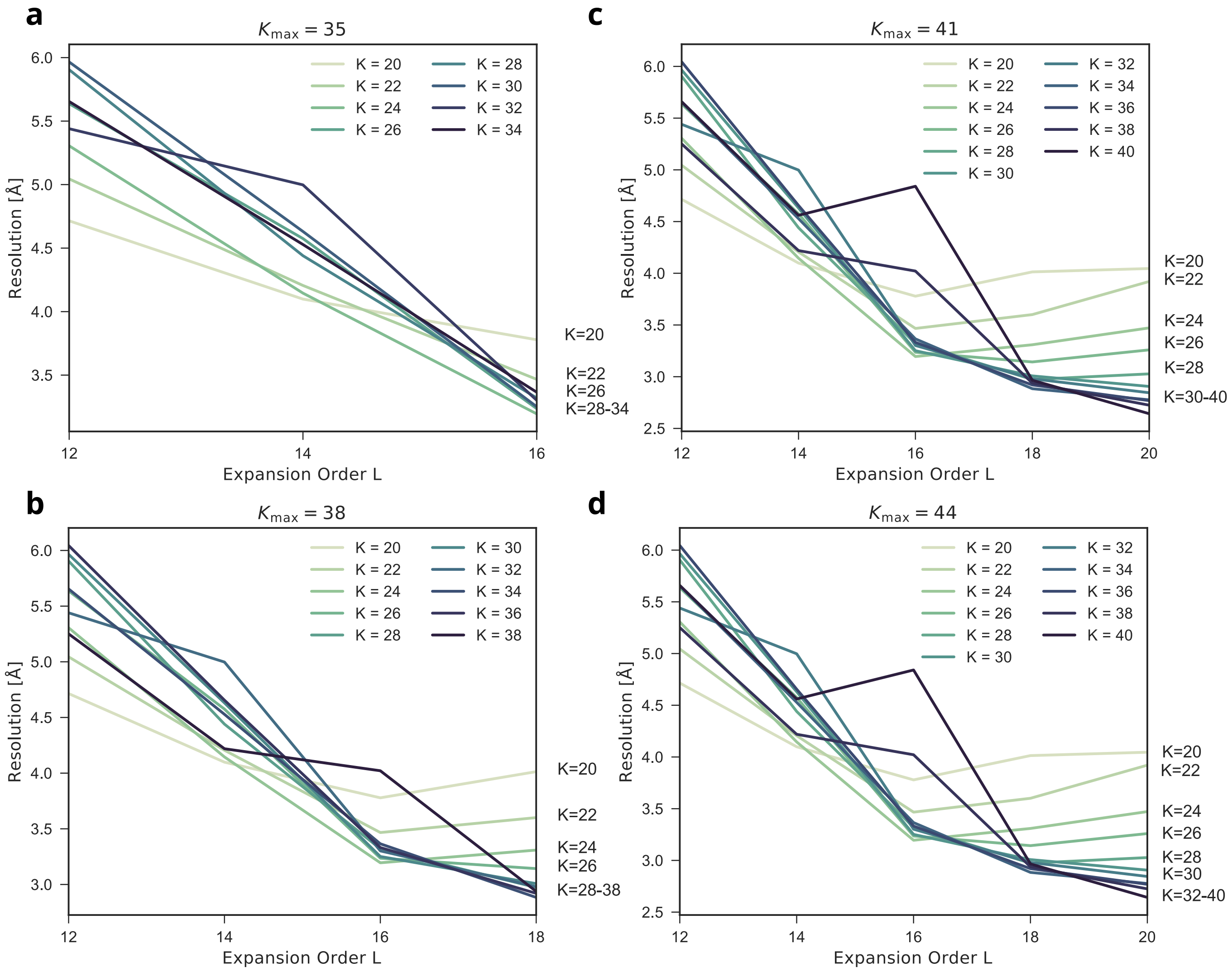

Supplementary Figure 2: Dependence of Resolution on SH-Parameters

Comparison of the effect on resolution of K_{max}, K and L for different parameter combinations. By increasing K_{max} (35(a), 38(b), 41(c), 43(d)), higher order terms in the spherical harmonics expansion and larger K result in increased resolution.

spacing Δk and the maximum expansion order $L_{\text{max}} = K_{\text{max}}/2$ to which the intensity model, used in the Monte Carlo search, is initially determined. The second parameter is the number of shells $K \leq K_{\text{max}}$ of the intensity model in the structure determination, which determines the maximum wave number $k_{\text{cut}} = K \cdot \Delta k$ and sets an upper bound for the resolution. The

third parameter is the expansion order $L \leq K_{\max}/2$ of the intensity model, which controls the angular resolution of the intensity model. The angular resolution of the intensity does not directly correspond to the resolution of the real-space electron density which is why the impact of L on the resolution is indirect. However, for each wave number k_{cut}, there is a minimum L that is required to describe the intensity sufficiently accurately.

Here, we aimed at the optimal set of parameters (K_{opt}, L_{opt}, $K_{\max,\text{opt}}$) by which a specific resolution is achieved with minimal computational effort (see Supplementary Note 2 for an estimate of the computational complexity). For our parameter optimization, we further assumed that an infinite number of photons is recorded up to the maximum wave number $k_{\max}$.

As an example, we aimed at a resolution of 3 Å. To determine the suitable parameters, we calculated the corresponding real space resolution of intensity models with varying expansion parameters K, L and $K_{\max}$. Supplementary Figure 2 shows the achieved resolution as a function of L for various number of shells K for four different $K_{\max}$ (35, 38, 41, 44). Note that the maximum possible L and K increases with $K_{\max}$ but due to the decrease of Δk the k_{cut} ($k_{\text{cut}} = K \cdot \Delta k$) of the model does not increase the same way. In all the cases, $L_{\text{opt}} = K_{\max}/2$ equalled the maximum possible expansion order and $K_{\max}$ and K were the limiting parameters.

From all parameter combinations yielding a resolution close to 3 Å, $K_{\max} = 38$, $K = 26$ and $L = 18$ minimized the computational effort, with the matrix multiplication of $\mathbf{A} \in \mathbb{R}^{163,153 \times 17,576}$ with $\mathbf{F} \in \mathbb{R}^{1024 \times 163,153}$ for each Monte Carlo step was the limiting factor. Several days were required for each structure determination run.

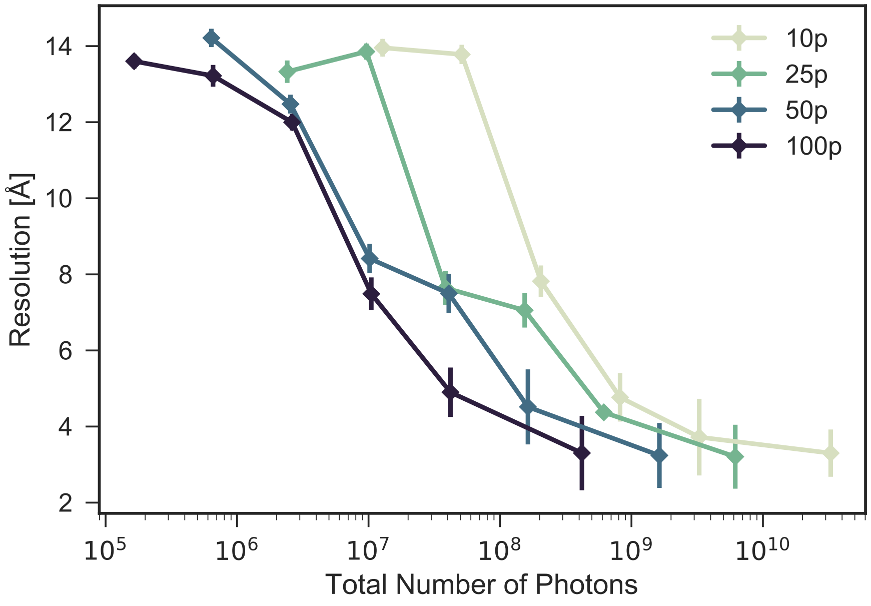

Supplementary Figure 3: Dependence of Resolution on Photons per Image

The resolution as a function of the total number of photons collected from images with 10, 25, 50 and 100 photons on average.

SUPPLEMENTARY NOTE 8: VARIATION OF THE PHOTON COUNTS PER IMAGE

In our histogram approach, the maximum number of triplets T that can be collected from an image with P photons is $T = P \cdot (P-1) \cdot (P-2)/6$. However, these triplets are not all statistically independent; rather, starting from 3 photons, each additional photon adds only two real numbers to the triple correlation: a new angle β (with respect to another photon) and a new distance k to the detector center.

The sampling of the three-photon correlation is improved by either collecting more photons per image P or by collecting more images I. However, because for each image, the orientation (3 Euler angles) needs to be inferred, the total amount of information that remains available

for structure determination increases with the number of photons per image. Therefore, for every structure determination method, including ours, increasing P is preferred over increasing I, especially at low photon counts. For larger photon counts, the ratio between the 3 Euler angles and P becomes small and hence also the information asymmetry between P and I.

To assess this effect, we asked how the resolution depends on the number of images I and the photons per image P and therefore carried out additional synthetic experiments using image sets with 10, 25, 50 and 100 average photons P per shot at different image counts yielding different total number of photons. In Supplementary Figure 3, the achieved resolutions are shown as a function of the number of collected photons for four different $P = [10, 25, 50, 100]$. For the best achievable resolution of 3.3 Å, e.g., the total number of required photons decreases by a factor of 100 from 3.3×10^{10} to 3.3×10^{8} photons (and the number of images decreased by a factor of 1000 from 3.3×10^{9} to 3.3×10^{6} images) when increasing the photons per image from 10 to 100, thus substantially decreasing the data acquisition time from over 20.000 minutes to only 30 minutes (see Fig. 3d main text).

SUPPLEMENTARY NOTE 9: STRUCTURE DETERMINATION IN THE PRESENCE OF ADDITIONAL NON-POISSONIAN NOISE

To asses how additional noise (beyond the Poisson noise due to low photon counts) affects the achievable resolution, we have carried out synthetic scattering experiments including Gaussian distributed photons, $G(\mathbf{k}, \sigma) = (2\pi\sigma^2)^{-1/2} \exp\left(-|\mathbf{k}|^2/2\sigma^2\right)$ (Supplementary Fig. 4), as a simple noise model. From the generated scattering images, intensities $S(\mathbf{k})$ were determined as described in the main text.

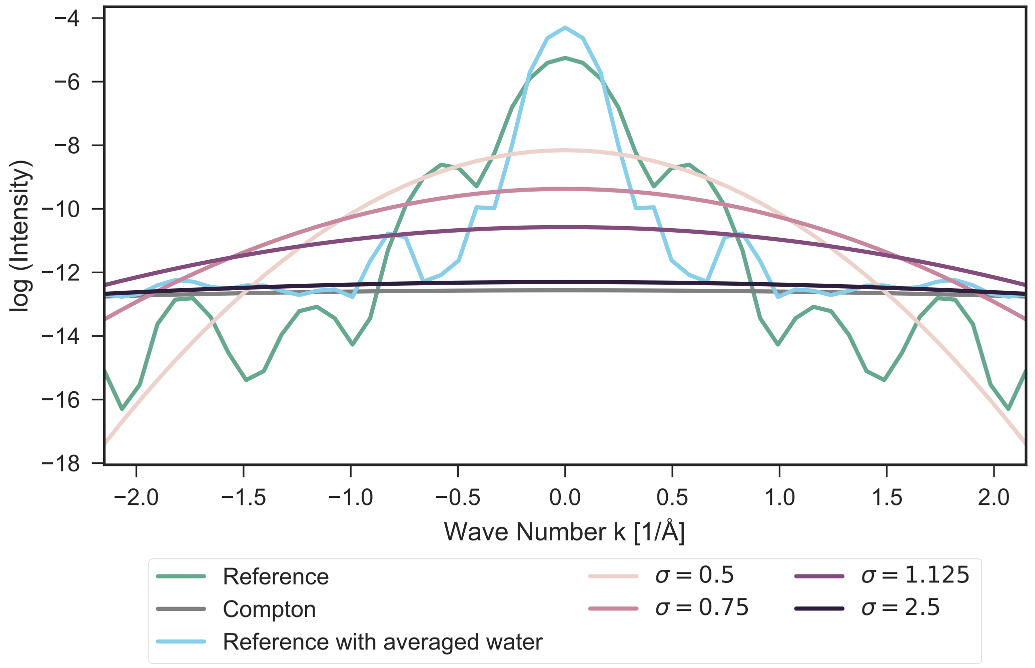

Supplementary Figure 4: Radial Intensity Distribution of Various Noise Sources

Comparison of linear cuts through the normalized intensities of noise distributed according to Gaussian functions with widths $\sigma = [0.5, 0.75, 1.125, 2.5]\,\text{Å}^{-1}$ (purple shades and black), noise from Compton scattering (grey) and noise from the a disordered water shell of 5 Å thickness (aqua). A cut through the Crambin intensity without noise (green) is given for reference. Note that, due to the normalization in 3D, the noise intensities are shown at a signal to noise ratio $\gamma = 100\%$; at different signal to noise ratios, the noise intensities are shifted vertically with respect to the Crambin intensity.

Assuming that the noise is independent of the molecular structure, the obtained intensities $S(\mathbf{k}) = I(\mathbf{k}) + \gamma N(\mathbf{k})$ are a linear superposition of the molecules' intensity $I(\mathbf{k})$ and the intensity of the unknown noise $N(\mathbf{k})$. Accordingly, the noise was subtracted from $S(\mathbf{k})$ in 3D Fourier space using our noise model $N(\mathbf{k}) = G(\mathbf{k}, \sigma)$ and the estimated signal to noise ratio γ. Since the spherical harmonics expansion of a Gaussian distribution is described by

a single coefficient $G_{l=0,m=0}(k) = G(k,\sigma)$ on each shell k, the noise subtraction simplified to $A^{\text{noise-free}}_{l=0,m=0}(k) = A^{\text{noisy}}_{l=0,m=0}(k) - \gamma G(k,\sigma)$. The noise-free intensity $I(\mathbf{k})$ was then processed as described in the main text.

As discussed in the main text, we assessed the effect of noise for different Gaussian widths ($\sigma = [0.5, 0.75, 1.125, 2.5]\,\text{Å}^{-1}$ and several signal to noise ratios $\gamma \in [10\%, ..., 50\%]$. Supplementary Figure 4 compares the Crambin intensity (green) with the different Gaussian distributions (puples shades,black) at signal to noise ratio of $\gamma = 100\%$.

The Figure also shows the noise expected from Compton scattering (grey), which was estimated using the Klein-Nishina differential cross-section [14]

$$\mathrm{d}\sigma = \frac{1}{2}\frac{\alpha^2}{m^2}\left(\frac{E'}{E}\right)^2\left[\frac{E'}{E} + \frac{E}{E'} - \sin^2\theta\right]\mathrm{d}\Omega, \tag{16}$$

with the scattering angle θ, the energy of the incoming photons E, the energy of the scattered photon $E' = E/(1+\frac{E}{m}(1-\cos\theta))$, the fine structure constant $\alpha = 1/137.04$ and the electron resting mass $m_\mathrm{e} = 511\ \text{keV}/c^2$. As can be seen, the noise from Compton scattering (grey) is described well by a Gaussian distributions with width $\sigma = 2.5\,\text{Å}^{-1}$ (black), and thus was used to approximate incoherent scattering.

Finally, we also estimated the noise from the disordered fraction of the water shell by averaging the intensities of 100 Crambin structures with different 5 Å-thick water shells. The resulting intensity (aqua) is similar to the reference intensity with fewer signal in the intermediate regions ($0.2\,\text{Å}^{-1} < k < 1.0\,\text{Å}^{-1}$) and more signal in the center and the high-resolution regions ($k > 1.0\,\text{Å}^{-1}$). Since the noise of the water shell depends on the structure of the biomolecule, potentially combined with ordered water molecules, it is unlikely to be well described by our simple Gaussian model. Therefore, simple noise subtraction will be challenging, and more advanced iterative techniques will be required.

SUPPLEMENTARY NOTE 10: PROCESSING EXPERIMENTAL SCATTERING IMAGES

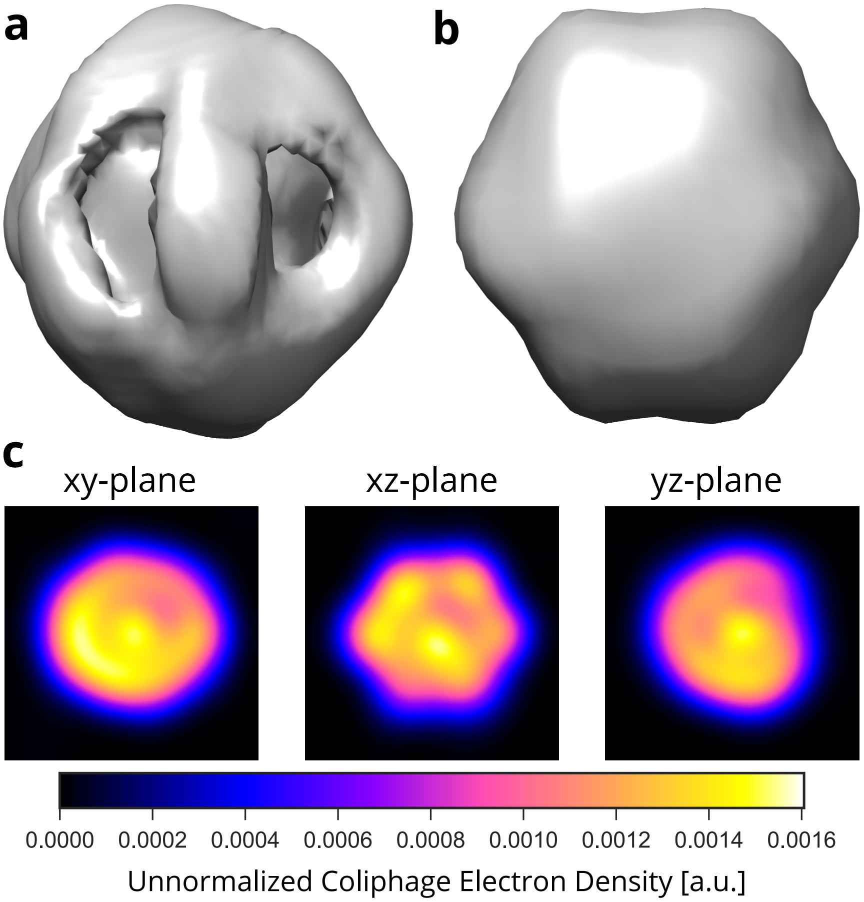

Supplementary Figure 5: Retrieved Intensity and Electron Density of a Coliphage Virus (a) Averaged intensity retrieved from 7350 images of the coliphage PR772 imageset [15] using three-photon correlations sampled with 3×10^{12} triplets. (b) Corresponding electron density after phase retrieval. (c) Three orthogonal planar slices through the retrieved electron density.

We have tested the structure determination with the coliphage PR772 image-set recorded at LCLS [15] to demonstrate that our method can handle the heterogeneities of real experimental data. The images contain a small beamstop which distorts the three-photon correlation

as follows,

$$t_{k_1,k_2,k_3,\alpha,\beta} = \left\langle \langle I_{\omega,\varphi}(\mathbf{k}_1) I_{\omega,\varphi}(\mathbf{k}_2) I_{\omega,\varphi}(\mathbf{k}_3)\rangle_\omega \right\rangle_\varphi \tag{17}$$

$$= \langle I_\omega(\mathbf{k}_1) I_\omega(\mathbf{k}_2) I_\omega(\mathbf{k}_3)\rangle_\omega \langle B_\varphi(\mathbf{k}_1) B_\varphi(\mathbf{k}_2) B_\varphi(\mathbf{k}_3)\rangle_\varphi .$$

Here, we described the underlying intensity $I_{\omega,\varphi}$ as the product of the full intensity $I_\omega(\mathbf{k})$ and the beamstop $B_\varphi(\mathbf{k})$. The second average over φ is only along a circle and expresses the three-photon correlation $b_{k_1,k_2,k_3,\alpha,\beta}$ of the beamstop only. The distortion of the two-photon correlation is similar and we have corrected both correlations with the respective beamstop correlations.

The available 14700 coliphage images contain over 400,000 photons per image on average. To demonstrate that our method can handle much fewer photons per image, we have down-sampled the images by generating individual photons, and triplets respectively, using rejection sampling (see Methods) of the intensity distribution given by the dense images. This allows us to generate, in principle, only three photons per image, however at a need for many more images. In order to achieve sufficient sampling with the limited number of images, we used 1200 photons per image on average, which is the same as reusing images multiple times. The specific number of photons in each image was scaled proportional to the integral over the entire image, i.e., the total number of scattered photons for each image. For the correlation we used $K = 38$ shells corresponding to $\Delta q = 0.004^{-1}$ and for the structure determination we used an expansion limit of $L = 12$. No symmetry was imposed on the intensity and the missing intensity in the center of the beamstop was completed using a fit of the adjacent shells with a Gaussian.

In order to calculate the resolution of the retrieved densities using Fourier shell correlations,

we calculated two independent two- and three-photon correlations from 7350 images of the coliphage virus each. From each of the two correlation, we determined 20 independent intensities and averaged them before phasing (see Supplementary Fig. 5a) and then also averaged 8 phased electron densities (see Supplementary Fig. 5b and c). Despite the large conformational inhomogeneities in the data set as mentioned by Hosseinizadeh *et al.* [16], the icosahedral symmetry of the virus is clearly visible in the xz-plane. Notably, this symmetry is a result of our reconstruction — in contrast to previous reconstructions [16], where the icosahedral symmetry of the particle was imposed. From the Fourier shell correlation

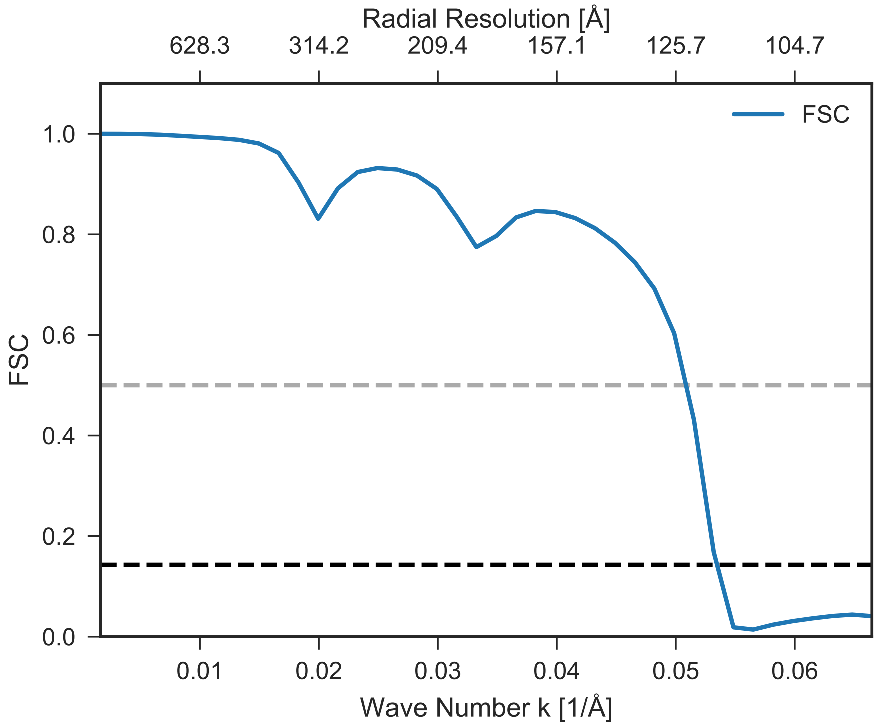

Supplementary Figure 6: Fourier Shell Correlation of the Retrieved Densities

Fourier shell correlation (FSC) between two independent structure determinations from 7350 images each. We achieved a resolution of 11.7 nm when using a standard 0.143 FSC cutoff and a 12.3 nm resolution using a 0.5 cutoff. The maximum achievable resolution based on the maximum scattering angle is 9 nm.

between the two retrieved electron densities, we calculated a resolution of 11.7 nm, which is close to the maximum achievable resolution of 9 nm (see FSC in Supplementary Fig. 6). We attribute the slightly lower resolution to the fact that we have not imposed any symmetry during reconstruction.

SUPPLEMENTARY NOTE 11: PROCESSING IMAGES WITH MULTIPLE PARTICLES

Structure determination approaches are usually limited by the total number of single molecule shots that can be recorded. Remarkably, our method can process images with multiple illuminated particles because the two- and three-photon correlations of these images are connected to the correlations of the single particle shots. In order to show this relation, here, we derive the connection for the two-particle case.

The intensity of an image containing two randomly oriented particles $I_2(\mathbf{k})$ is the superposition of the the individual particle intensities' with the relative orientation being random,

$$\begin{aligned} I_2(\mathbf{k}) &= \langle I(\mathbf{k}) + I_\omega(\mathbf{k})\rangle_\omega \\ &= I(\mathbf{k}) + \langle I_\omega(\mathbf{k})\rangle \\ &= I(\mathbf{k}) + I^1(k). \end{aligned} \tag{18}$$

The two-photon correlation then reads,

$$\begin{aligned} c^{(2)}_{k_1,k_2,\alpha} &= \langle I_2(\mathbf{K}_1) I_2(\mathbf{K}_2)\rangle >_\omega \\ &= \big\langle I(\mathbf{K}_1)I(\mathbf{K}_2) + I(\mathbf{K}_1)I^1(k_2) + I^1(k_1)I(\mathbf{K}_2) + I^1(k_1)I^1(k_2)\big\rangle >_\omega \\ &= c^{(1)}_{k_1,k_2,\alpha} + 3I^1(k_1)I^1(k_2) \end{aligned} \tag{19}$$

and the three-photon correlation of the two-particle case is calculated as,

$$
\begin{aligned}
t^{(2)}_{k_1,k_2,k_3,\alpha,\beta} &= \langle I_2(\mathbf{K}_1) I_2(\mathbf{K}_2) I_2(\mathbf{K}_3)\rangle_\omega \\
&= \left\langle (I(\mathbf{K}_1) + I^1(k_1))(I(\mathbf{K}_2) + I^1(k_2))(I(\mathbf{K}_3) + I^1(k_3))\right\rangle_\omega \\
&= < I(\mathbf{K}_1)I(\mathbf{K}_2)I(\mathbf{K}_3) + I^1(k_1)I(\mathbf{K}_2)I(\mathbf{K}_3) + I(\mathbf{K}_1)I^1(k_2)I(\mathbf{K}_3) + I(\mathbf{K}_1)I(\mathbf{K}_2)I^1(k_3) + \\
&\quad \times I^1(k_1)I^1(k_2)I(\mathbf{K}_3) + I^1(k_1)I(\mathbf{K}_2)I^1(k_3) + I(\mathbf{K}_1)I^1(k_2)I^1(k_3) + I^1(k_1)I^1(k_2)I^1(k_3) >_\omega \\
&= t^{(2)}_{k_1,k_2,k_3,\alpha,\beta} + I^1(k_2)c^{(1)}_{k_1,k_3,\beta} + I^1(k_1)c^{(1)}_{k_2,k_3,(\alpha-\beta)} + I^1(k_3)c^{(1)}_{k_1,k_2,\alpha} + 4I^1(k_1)I^1(k_2)I^1(k_3)
\end{aligned} \tag{20}
$$

The expressions above is readily generalize to the N-particle case and the only remaining unknowns are the mixture ratios γ_i for the N_i-particles, i.e. the fraction of images containing N_i particles. These ratios are equivalent to the ratios between the integrated intensities of the individual images which identifies the total number of particle in each image and therefore can be calculated from the experimental data without additional effort.

SUPPLEMENTARY NOTE 12: NOTE ON EWALD CURVATURE

In the initial version of this paper, the computationally expensive structure determination runs were carried out with a planar approximation of the Ewald sphere, i.e., $\lambda = 0$ Å. However, we expected that the structure results would only slightly change in presence of the Ewald curvature, because the entire Fourier intensity is still fully sampled. With the inclusion of the Ewald curvature in both the theory and the implementation of the algorithms, as presented in this paper, we have re-performed the synthetic scattering experiments at a beam wavelength of 2.5 Å and determined the structure from both 3.3×10^9 and 3.3×10^8 images. For our maximum wave number of $k_{\max}$ the curvature of the Ewald sphere leads to a deviation from the plane by $\theta = 65°$. Both structure determination runs gave similar resolutions as for the planar case (3.3 Å and 3.7 Å) and we concluded that the results

presented in this paper are not affected by the additional Ewald curvature.

SUPPLEMENTARY FIGURES

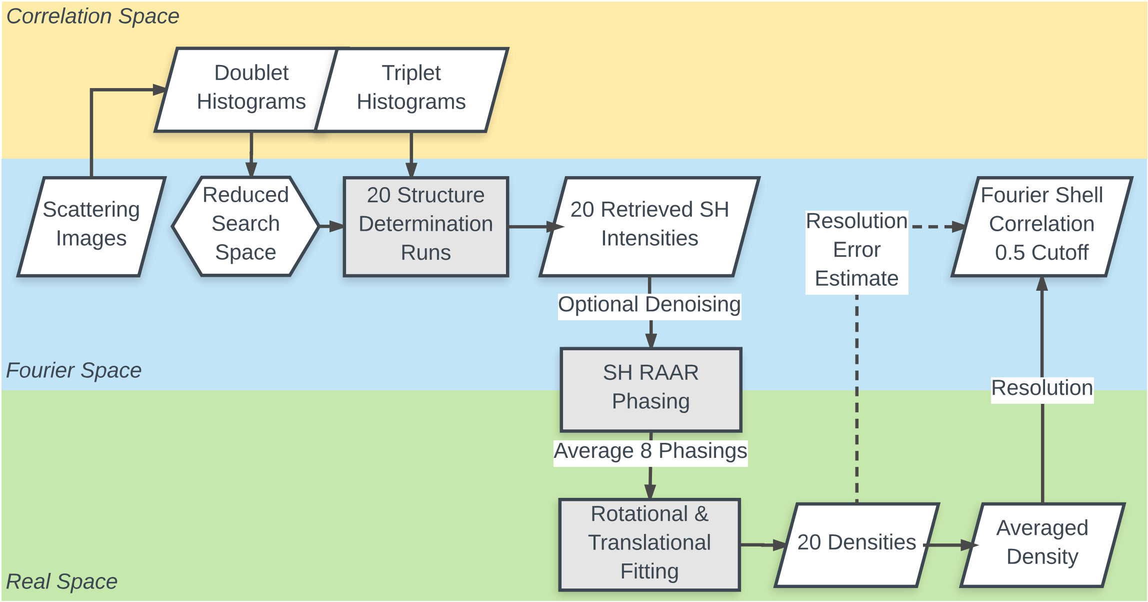

Supplementary Figure 7: Calculation of Electron Densities and Resolution

Flowchart outlining how electron densities are calculated from the scattering images. The resolution and its error is calculated from the average of 20 phased electron densities.

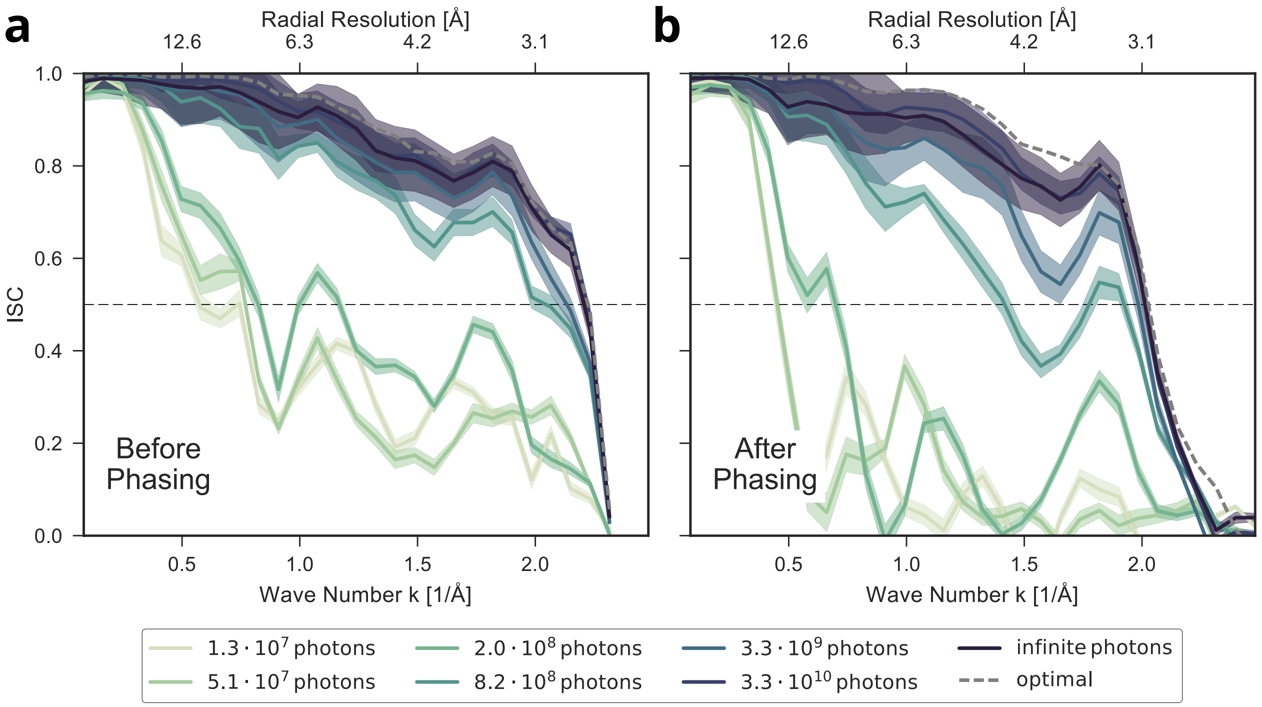

Supplementary Figure 8: Evaluation of Phasing Errors for Crambin

Comparison between the intensity shell correlation (ISC) of the retrieved intensities before the phasing was done (a) and the ISC calculated from the phased electron densities (b). The 0.5 cutoff is given to estimate the quality of the respective intensites. The phasing leads to a moderate decrease of the threshold-crossing by ca. 0.3 Å.

SUPPLEMENTARY REFERENCES

[1] Kam, Z. The reconstruction of structure from electron micrographs of randomly oriented particles. *Journal of Theoretical Biology* **82**, 15–39 (1980).

[2] Baddour, N. Operational and convolution properties of three-dimensional Fourier transforms in spherical polar coordinates. *Journal of the Optical Society of America A* **27**, 2144 (2010).

[3] Wigner, E. P. On the Matrices Which Reduce the Kronecker Products of Representations of S. R. Groups. In *Quantum Theory of Angular Momentum*, 87–133 (Springer Berlin Heidelberg,

Berlin, Heidelberg, 1965).

[4] Kostelec, P. J., Maslen, D. K., Healy Jr., D. M. & Rockmore, D. N. Computational Harmonic Analysis for Tensor Fields on the Two-Sphere. *Journal of Computational Physics* **162**, 514–535 (2000).

[5] Skibbe, H., Wang, Q., Ronneberger, O., Burkhardt, H. & Reisert, M. Fast computation of 3D spherical Fourier harmonic descriptors - A complete orthonormal basis for a rotational invariant representation of three-dimensional objects. *2009 IEEE 12th International Conference on Computer Vision Workshops, ICCV Workshops 2009* 1863–1869 (2009).

[6] Wieder, T. A generalized Debye scattering formula and the Hankel transform. *Zeitschrift fur Naturforschung - Section A Journal of Physical Sciences* **54**, 124–130 (1999).

[7] Toyoda, M. & Ozaki, T. Fast spherical Bessel transform via fast Fourier transform and recurrence formula. *Computer Physics Communications* **181**, 277–282 (2010).

[8] Yu, L. *et al.* Quasi-discrete Hankel transform. *Optics letters* **23**, 409–411 (1998).

[9] Blanco, M. a., Flórez, M. & Bermejo, M. Evaluation of the rotation matrices in the basis of real spherical harmonics. *Journal of Molecular Structure: THEOCHEM* **419**, 19–27 (1997).

[10] Homeier, H. H. & Steinborn, E. Some properties of the coupling coefficients of real spherical harmonics and their relation to Gaunt coefficients. *Journal of Molecular Structure: THEOCHEM* **368**, 31–37 (1996).

[11] Stuhrmann, H. B. Interpretation of small angle scattering functions of dilute solutions and gases. *Acta Crystallographica Section A* **26**, 297–306 (1970).

[12] Mezzadri, F. How to generate random matrices from the classical compact groups. *Preprint at http://arXiv.org/math-ph/0609050* (2006). Preprint at http://arXiv.org/math-ph/0609050.

[13] Rosenthal, J. S. Random rotations: characters and random walks on SO(N). *The Annals of Probability* **22**, 398–423 (1994).

[14] Klein, O. & Nishina, T. Über die Streuung von Strahlung durch freie Elektronen nach der neuen relativistischen Quantendynamik von Dirac. *Zeitschrift für Physik* **52**, 853–868 (1929).

[15] Reddy, H. K. N. Data Descriptor: Coherent soft X-ray diffraction imaging of coliphage PR772 at the Linac coherent light source Background & Summary. *Scientific data* **4**, 170079 (2017).

[16] Hosseinizadeh, A. *et al.* Conformational landscape of a virus by single-particle X-ray scattering. *Nature Methods* **14**, 877–881 (2017).
